# Intractable Recurrent Abscess Around the Nipple Caused by Mastitis Remitted by Kampo Medicine Treatment: A Case Report

**DOI:** 10.7759/cureus.78105

**Published:** 2025-01-27

**Authors:** Shin Takayama, Tsukushi Kaji, Akihiko Furuta, Kaolu Sato

**Affiliations:** 1 Department of Education and Support for Regional Medicine, Tohoku University Hospital, Sendai, JPN; 2 Department of Breast Surgery, Japanese Red Cross Ishinomaki Hospital, Ishinomaki, JPN

**Keywords:** abscess, kampo medicine, mastitis, nipples, ultrasonography

## Abstract

We present a case of a 46-year-old woman with recurrent breast abscess resistant to conventional treatments. Initial diagnosis of mastitis led to antibiotic therapy; however, abscess formation recurred. Subsequent interventions, including incision, drainage, and various antibiotics, were insufficient because of recurrent infections. Due to the side effects of long-term antibiotic use, the patient was referred to the Kampo medicine department to address the premenstrual symptoms and recurrent infections. Treatment with Kampo medicines resulted in significant symptom reduction within a month. After treatment, the patient experienced mild symptoms, and breast abscess recurrence was prevented for more than two years. This case highlights the potential role of Kampo medicine in the management of refractory breast abscesses associated with premenstrual symptoms. Further research is required to explore the efficacy and mechanisms of action of Kampo medicines in similar cases.

## Introduction

Mastitis causes inflammation in the udder tissue [1] and is a multifactorial disease caused by various bacterial, viral, and fungal agents that invade the mammary gland, resulting in inflammation [2]. It has both acute and chronic phases, and recurrent cases are sometimes intractable [3]. Recurrent mastitis is commonly associated with breastfeeding but also occurs in non-lactating individuals. It is characterized by symptoms such as breast pain, redness, swelling, warmth, and sometimes fever. Causes of recurrent mastitis are incomplete milk drainage, bacterial infection, nipple damage, immune conditions, smoking, and previous infections. Management of recurrent mastitis involves proper breastfeeding, nipple and breast hygiene, antibiotic treatment, and avoiding mechanical pressure on the breasts. Frequent use of antimicrobial agents increases the likelihood of drug resistance and should be avoided from the perspective of antimicrobial resistance (AMR). Thus, the control of infection and inflammation in recurrent mastitis with Kampo medicines will contribute to reduced AMR.

A multidisciplinary approach that suppresses relapse and worsening is key for chronic stage control. Kampo medicine is a traditional Japanese medicine that involves herbal medicines and/or acupuncture for treatment. The original concept was transferred from ancient China almost 1500 years ago [4]. Kampo medicines are composed of several crude drugs, combined based on historical knowledge. Kampo specialty doctors prescribe these medicines according to symptoms, conditions, and examinations, including tongue, pulse, and abdominal examinations.

Herein, we report a case of an intractable recurrent abscess around the nipple caused by mastitis that was remitted by Kampo medicine treatment.

## Case presentation

**Figure 1:**
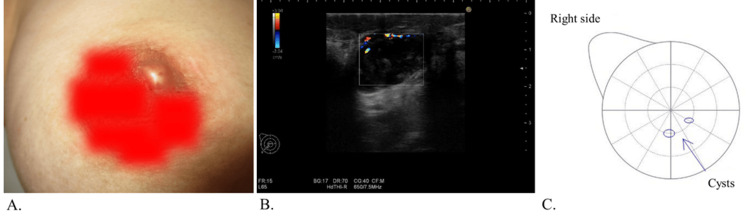
(A) Abscess formation around the right nipple. (B) Ultrasonography of the right nipple revealed abscess formation with a blood flow signal. (C) Ultrasonography imaging around the right nipple after drainage and antibiotic administration showed no abscess formation, but several existing cysts.

A 46-year-old woman with a history of multiple cysts in both breasts complained of right breast pain and swelling around the right nipple and visited our hospital in January 2020. She had no history of cancer, diabetes mellitus, collagen diseases, or mechanical injury to the chest. She did have a history of one pregnancy and one childbirth. Mastitis was suspected, and amoxicillin/clavulanic acid antibiotics were prescribed. Figure 1 illustrates the timeline of treatment and symptom resolution. A follow-up medical checkup after treatment revealed that her symptoms persisted, and abscess formation around the right nipple was confirmed (Figure 1A). Ultrasonography of the right nipple revealed the formation of an abscess with a blood flow signal in the upper exterior area, and the patient was diagnosed with a submammary abscess (Figure 1B). The patient underwent incision and drainage, followed by amoxicillin/clavulanic acid administration. Leachate culture was positive for *Staphylococcus capitis* (2+) and *Corynebacterium* species (3+).

Although the symptoms initially resolved, the patient reported itching and swelling around the right nipple two weeks after the final administration of antibiotics. Amoxicillin/clavulanic acid was prescribed again; however, two weeks later, ultrasonography revealed abscess reformation. The physician then performed puncture and drainage, followed by the administration of levofloxacin, which was effective in bacterial culture. She complained of breast swelling and pain accompanying menstrual cycles, and the abscess recurred each time. Multiple puncture drainages and antimicrobial treatments for recurrent infection and abscess formation were performed, but the infection was not controlled. Repeated abscess cultures showed amoxicillin/clavulanic acid- and levofloxacin-resistant *Corynebacterium* (2+); thus, sulfamethoxazole/trimethoprim was selected as the next antibiotic. However, the infection was not controlled by the administration of sulfamethoxazole/trimethoprim. The physician consulted the appropriate antimicrobial team and recommended long-term administration of minocycline. The administration of minocycline for seven months suppressed the infection (Figure 1C); however, blood tests revealed liver dysfunction; thus, minocycline was discontinued. The infection recurred four months later, and the patient was again administered minocycline. Symptoms of swelling and pain were observed, but the patient complained of general fatigue after minocycline administration. Gamma-glutamyl transpeptidase (γ-GTP) increased from 47 U/L to 51 U/L after minocycline administration, and antibiotic side effects were suspected.

The symptoms and infections were intractable and recurrent, and the patient was referred to the Kampo Medicine Department for the control of premenstrual syndrome and recurrent infection around the right nipple. Physical data showed a 36.7℃ body temperature, 155.5 cm body height, 58 kg body weight, 138/89 mmHg blood pressure, and 90 bpm pulse. Laboratory data from the first visit to the Kampo Medicine Department are presented in Table 1. The clinical course with conventional and Kampo treatment is shown in Table 2.

**Table 1:** Laboratory data at first visit to the Kampo Medicine Department. AST: aspartate aminotransferase; ALT: alanine aminotransferase; LDH: lactate dehydrogenase; ALP: alkaline phosphatase; γ-GTP: gamma-glutamyl transpeptidase; ChE: cholinesterase; T-Cho: total cholesterol; BUN: blood urea nitrogen; Cr: creatinine; UA: uric acid; Na: sodium; K: potassium.

Test	Result	Unit	Interpretation	Reference range
Total protein	6.9	g/dL	Normal	6.6-8.1
AST	24	U/L	Normal	13-30
ALT	17	U/L	Normal	7-23
LDH	191	U/L	Normal	124-222
ALP	90	U/L	Normal	38-113
γ-GTP	47	IU/L	High	9-32
ChE	264	U/L	Normal	201-421
T-Cho	176	mg/dL	Normal	142-248
BUN	17	mg/dL	Normal	8-20
Cr	0.76	mg/dL	Normal	0.46-0.79
UA	5.4	mg/dL	Normal	2.6-5.5
Na	139	mEq/L	Normal	138-145
K	4.4	mEq/L	Normal	3.6-4.8

**Table 2:** Clinical course with conventional treatment and Kampo treatment. ● indicates the event held that month.

	2020												2021												2022											2023													2024			
	1	2	3	4	5	6	7	8	9	10	11	12	1	2	3	4	5	6	7	8	9	10	11	12	1	2	3	4	5	6	7	8	9	10	11	12	1	2	3	4	5	6	7	8	9	10	11	12	1	2	3	4
Breast swelling	●	●	●	-	●	●	-	●	-	●	-	●	-	●	●	-	-	-	-	-	-	-	-	-	-	●	-	-	●	-	-	-	-	-	-	●	●	-	-	●	●	-	●	-	-	-	-	●	●	-	-	-
Abscess formation	-	●	●	-	-	●	-	●	-	●	-	●	-	●	●	-	-	-	-	-	-	-	-	-	-	●	-	-	-	-	-	-	-	-	-	-	-	-	-	-	-	-	-	-	-	-	-	-	-	-	-	-
Incision or puncture to drain pus	-	●	●	-	-	●	-	●	-	●	-	●	-	●	●	-	-	-	-	-	-	-	-	-	-	●	-	-	-	-	-	-	-	-	-	-	-	-	-	-	-	-	-	-	-	-	-	-	-	-	-	-
Antibiotics																																																				
Amoxicillin/clavulanic acid	●	●	●	-	-	●	-	-	-	-	-	-	-	-	-	-	-	-	-	-	-	-	-	-	-	-	-	-	-	-	-	-	-	-	-	-	-	-	-	-	-	-	-	-	-	-	-	-	-	-	-	-
Levofloxacin	-	-	●	-	●	-	-	-	-	-	-	-	-	-	-	-	-	-	-	-	-	-	-	-	-	-	-	-	-	-	-	-	-	-	-	-	-	-	-	-	-	-	-	-	-	-	-	-	-	-	-	-
Sulfamethoxazole/trimethoprim	-	-	-	-	-	-	-	●	-	●	-	●	-	●	-	-	-	-	-	-	-	-	-	-	-	-	-	-	-	-	-	-	-	-	-	-	-	-	-	-	-	-	-	-	-	-	-	-	-	-	-	-
Minocycline	-	-	-	-	-	-	-	-	-	-	-	-	-	-	●	●	●	●	●	●	●	-	-	-	-	●	-	-	-	-	-	-	-	-	-	-	-	-	-	-	-	-	-	-	-	-	-	-	-	-	-	-
Kampo medicines																																																				
Kamishoyosan and goreisan	-	-	-	-	-	-	-	-	-	-	-	-	-	-	-	-	-	-	-	-	-	-	-	-	-	-	-	-	●	●	●	●	●	●	●	●	●	●	●	●	●	●	●	●	●	●	●	●	●	●	●	●
Hainosankyuto	-	-	-	-	-	-	-	-	-	-	-	-	-	-	-	-	-	-	-	-	-	-	-	-	-	-	-	-	●	-	-	-	-	-	-	●	●	-	-	●	●	-	●	-	-	-	-	●	●	-	-	-

The patient complained of hot flashes, headache, dizziness due to atmospheric fluctuations, breast tension associated with menstruation, and menstrual blood clots. We prescribed 2.5 g of kamishoyosan (TSUMURA & Co., Tokyo, Japan) [5] three times per day for the premenstrual syndrome of hot flashes and breast tension. Additionally, 2.5 g of goreisan (TSUMURA & Co., Tokyo, Japan) [5] three times per day was prescribed for headaches and dizziness caused by atmospheric fluctuations. Within a month, her symptoms were reduced as per the following observations on the numeric rating scale: hot flashes, 5 to 0; headache and dizziness, 10 to 2; and breast tension associated with menstruation, 10 to 2. In cases of signs of infection, such as slight swelling or itching, 2.5 g of hainosankyuto (TSUMURA & Co., Tokyo, Japan) [5] three times per day was administered for three days for temporary use. After Kampo medicine treatment, she experienced slight symptoms in her breast, but abscess formation has not recurred in over two years.

Kampo medicines

Kampo medicines, including components and indications [5], are shown in Table 3. Kamishoyosan is composed of 10 crude drugs. It is indicated for the relief of the following symptoms in women with a delicate constitution who are easily fatigued and are apt to have shoulder stiffness, psychoneurotic symptoms, including anxiety, and a tendency to constipation: oversensitivity to cold, delicate constitution, menstrual irregularity, dysmenorrhea, climacteric disturbances, and automatic imbalance syndrome peculiar to women resembling climacteric disturbance. Goreisan is composed of five crude drugs. It is indicated for the relief of the following symptoms in patients with oral dryness and decreased urine volume: edema, nephrosis, alcoholic hangover, acute gastrointestinal catarrh, diarrhea, nausea, vomiting, dizziness, water retention in the stomach, headache, uremia, heat-stroke, and diabetes mellitus. Hainosankyuto is composed of six crude drugs. It is indicated for the relief of symptoms of purulence with a reddened, swollen, and painful lesion, carbuncle, furuncle, facial furuncle, and other furunculosis.

**Table 3:** List of components and indications of Kampo medicines in the present study: kamishoyosan for pre-menstrual symptoms, goreisan for edema, and hainosankyuto for anti-inflammatory effects. Composition and indications are referred from reference [5]. JP: The Japanese Pharmacopoeia.

	Composition	Indications
Kamishoyosan	3.0 g of JP Bupleurum root, 3.0 g of JP peony root, 3.0 g of JP Atractylodes lancea rhizome, 3.0 g of JP Japanese angelica root, 3.0 g of JP Poria sclerotium, 2.0 g of JP Gardenia fruit, 2.0 g of JP Moutan bark, 1.5 g of JP Glycyrrhiza, 1.0 g of JP ginger, 1.0 g of JP Mentha herb.	Indicated for the relief of the following symptoms of those women with delicate constitutions who are easily fatigued and are apt to have stiffness shoulder, psychoneurotic symptoms, including anxiety, and sometimes tendency to constipation: oversensitivity to cold, delicate constitution, menstrual irregularity, dysmenorrhea, climacteric disturbance, and automatic imbalance syndrome peculiar to women resembling climacteric disturbance.
Goreisan	4.0 g of JP Alisma rhizome, 3.0 g of JP Atractylodes lancea rhizome, 3.0 g of JP Polyporus sclerotium, 3.0 g of JP Poria sclerotium, 1.5 g of JP cinnamon bark.	Indicated for the relief of the following symptoms of those patients with oral dryness and decreased urine volume: edema, nephrosis, alcoholic hangover, acute gastrointestinal catarrh, diarrhea, nausea, vomiting, dizziness, water retention in the stomach, headache, uremia, heat-stroke, and diabetes mellitus.
Hainosankyuto	4.0 g of JP Platycodon root, 3.0 g of JP Glycyrrhiza, 3.0 g of JP immature orange, 3.0 g of JP peony root, 3.0 g of JP jujube, 1.0 g of JP ginger.	Indicated for the relief of the symptoms of purulence with a reddened, swollen, and painful lesion, carbuncle, furuncle, facial furuncle, and other furunculosis.

## Discussion

Mastitis commonly affects breastfeeding women. Breast milk stasis and nipple injuries during breastfeeding increase the likelihood of bacterial infections. Mastitis can also occur in women who are not breastfeeding. Around the time of menopause, changes in hormone balance cause the mammary glands to atrophy, making it easier for blockages and inflammation to occur in the milk ducts. It can also occur in middle-aged and older women who smoke, have a weakened immune system, or have an underlying disease such as diabetes. Our case did not smoke or have a history of immunocompromise and mechanical injury to the chest.

After Kampo medicine treatment, the intractable recurrent abscesses around the nipple accompanied by mastitis were remitted. In this case, the pathogenic bacteria showed resistance to multiple antibiotics, and minocycline was selected for long-term administration. However, owing to the side effects of liver dysfunction and fatigue, antibiotics had to be discontinued. Symptoms of swelling, itching, and pain in the breast occurred during the menstrual cycle, and premenstrual symptoms needed to be controlled.

The Kampo medicine kamishoyosan is used to treat premenstrual symptoms, while goreisan is used for headaches, swelling, and atmospheric changes. The prescription of kamishoyosan with goreisan can suppress premenstrual symptoms and local skin edema. For signs of infection, such as slight swelling or itching, hainosankyuto is temporarily administered for three days. Kampo medicine has strong anti-inflammatory effects and is used to treat perianal abscesses. It is also used to treat mastitis and breast swelling.

Kampo medicines are widely prescribed by the Japanese health insurance system for women’s healthcare [6]. Nowadays, Kampo medicines are used by over 86% of Japanese physicians [7]. Kamishoyosan is the most frequently used Kampo medicine for the treatment of premenstrual syndrome and dysphoric disorder [8]. It is reported to reduce the concentrations of interleukin (IL)-6, macrophage inflammatory protein-1β, and IL-8 in women with hot flashes [9]. Yokukansan is also used for the treatment of premenstrual syndrome and dysphoric disorder, especially suppressing irritation by controlling the serotonin and glutamate network in the brain [10]. On the other hand, hangekobokuto is used for depressive symptoms and pharyngeal discomfort by controlling dopamine and noradrenalin levels in the brain [11]. Goreisan is used to control fluid retention [12], as it can increase the expression of vascular endothelial growth factor receptor-3 in lymphatic vessels [13], regulate aquaporin 3 expression [14], and inhibit aquaporin 4 function [15]. Hainosankyuto is used to treat perianal abscesses [16,17], and treatment is reported to be more beneficial than incision drainage [18]. Additionally, it enhances phagocytic activity, increases mRNA levels of IL-12 and interferon-C, and decreases mRNA levels of tumor necrosis factor-a [19]. It is also applied for the treatment of subperiosteal abscesses [20].

Based on basic and clinical effects, regular administration of kamishoyosan relieved premenstrual symptoms, including breast tension, whereas regular goreisan administration suppressed edema. Combining regular administration with temporary use of Kampo medicines enhanced their anti-inflammatory effects and provided symptom relief.

## Conclusions

Mastitis with abscess formation can recur. Antibiotic resistance and the side effects of long-term antibiotic use cause mastitis to be intractable. We reported a case of an intractable recurrent abscess around the nipple, which was remitted by Kampo medicine treatment. Kamishoyosan was used to relieve premenstrual symptoms, including breast tension, and hainosankyuto was used for its anti-inflammatory effects. Kampo medicines may successfully treat conditions that are intractable to Western medicine. In cases of recurrent mastitis, Kampo medicine treatment may be used for disease control.
